# Extinguishing the flames of inflammation: retardant effect of chlorquinaldol on NLRP3-driven diseases

**DOI:** 10.1186/s10020-024-01016-1

**Published:** 2024-12-19

**Authors:** Zhilei Wang, Jingwen Liu, Yu Mou, Yuchen Li, Wenhao Liao, Menglin Yao, Ting Wang, Hongping Shen, Qin Sun, Jianyuan Tang

**Affiliations:** 1 TCM Regulating Metabolic Diseases Key Laboratory of Sichuan Province, Hospital of Chengdu University of Traditional Chinese Medicine, Chengdu, 610075 China; 2 Hospital of Chengdu University of Traditional Chinese Medicine, Chengdu, 610075 China; 3 National Traditional Chinese Medicine Clinical Research Base of the Affiliated Traditional Chinese Medicine Hospital of Southwest Medical University, Luzhou, 646000 China

**Keywords:** Chlorquinaldol, NLRP3 inflammasome, Pyroptosis, Drug repurposing, Peritonitis, Colitis, Gouty arthritis

## Abstract

**Background:**

NLRP3 inflammasome immoderate activation results in the occurrence of various inflammatory diseases, but the clinic medications targeting NLRP3 inflammasome are still not available currently. The strategy of drug repurposing can reorient the direction of therapy, which is an indispensable method of drug research. In this study, an antimicrobial agent chlorquinaldol (CQ) was conducted to assess the effect on NLRP3 inflammasome and novel clinical value on NLRP3-driven diseases.

**Methods:**

The effect of CQ on NLRP3 inflammasome activation and pyroptosis was studied in mouse and human macrophages. ASC oligomerization, intracellular potassium, reactive oxygen species production, and NLRP3-ASC interaction were used to evaluate the suppression mechanism of CQ on inflammasome activation. Finally, the ameliorative effects of CQ in the model of LPS-induced peritonitis, dextran sodium sulfate (DSS)-induced colitis, and monosodium urate (MSU)-induced gouty arthritis were evaluated in vivo.

**Results:**

CQ is a highly powerful NLRP3 inhibitor that has feeble impact on the NLRC4 or AIM2 inflammasome activation in mouse and human macrophages. Further study indicated that CQ exhibits its suppression effect on NLRP3 inflammasome by blocking NLRP3-ASC interaction and hydroxyl on the benzene ring is vital for the assembly and activation of NLRP3 inflammasome. Furthermore, in vivo experiments demonstrated that administration of CQ has outstanding therapeutic action on LPS-induced peritonitis, DSS-induced colitis, and MSU-induced gouty inflammation in mice.

**Conclusions:**

Collectively, the current study discoveries the antimicrobial agent CQ as a potentially specific NLRP3 inhibitor, and its use provides a feasible therapeutic approach for the treatment of NLRP3-driven diseases.

## Introduction

Innate immunity serves as a first line of defense against danger signals, the invasion of exogenous pathogens and microbes. The innate immune response targets pathogen-associated molecular patterns (PAMPs) and damage-associated molecular patterns (DAMPs), which are identified by pattern recognition receptors (PRRs). The intracellular sensor NOD-like receptor (NLR) family, pyrin domain-containing-3 (NLRP3) is one of the most important PRRs in cytoplasm that detects extensive microbial sequences, endogenous/exogenous danger signals and environmental irritants (Wada and Makino 2016). NLRP3 responds to the signal by forming a complex with apoptosis-associated speck-like protein containing a CARD (ASC) and caspase 1 (Elliott and Sutterwala 2015; Wang et al. 2019a, 2020). After the assembly of inflammasome complex is completed, caspase 1 is self-cleave to form the active caspase 1, which can shear pro-IL-1β and pro-IL-18 to the active forms. Simultaneously active caspase 1 will cleave the protein Gasdermin D (GSDMD) into C-GSDMD and N-GSDMD. N-GSDMD migrates and anchors to the cell membrane, forming a pore on the cell membrane to initiate pyroptosis and trigger the secretion of active IL-1β and IL-18 (Huang et al. 2021; Paik et al. 2021; Wang and Hauenstein 2020). NLRP3 inflammasome aberrant activation leads to excessive inflammatory response, which is widely involved in the progression of serious diseases, such as inflammatory diseases, cardiovascular diseases, autoimmune diseases, neurodegenerative diseases, psychiatric diseases (Sharma and Kanneganti 2021; Strowig et al. 2012; Toldo et al. 2022). Therefore, the development of novel and effective NLRP3 inflammasome inhibitors is of great clinical significance.

New drug development process is characterized by large investment, small output, high risk and long time to market. Some drugs may also be withdrawn from the market due to adverse reactions, which not only bears huge economic losses, but also seriously threatens the life safety of patients. Drug repurposing is based on the reorientation of known drugs, which can not only save the time and cost of drug research and development, but also expand the scope of drug application, and make a horizontal comparison of the mechanism of action of different drugs (Parvathaneni et al. 2019; Pushpakom et al. 2019). Most of the drugs with new use and development value of old drugs have passed the pre-clinical and clinical safety evaluation. From the perspective of safety, the risk is lower than that of completely new drugs. Using the strategy of drug repurposing, the research and development efficiency can be greatly improved, and even can be directly applied to clinical emergency and experimental research. Especially in this COVID-19 epidemic, drug repurposing has become an important means and strategy of SARS-CoV-2 infection drug research and development, and has shown outstanding advantages in clinical treatment (Asselah et al. 2021; Ciliberto et al. 2020; Dotolo et al. 2021). Therefore, drug repurposing will become an important development direction of drug research and development in the future.

Chloroquinaldol (CQ), as a derivative of 8-hydroxyquinoline, is a long-term topical drug used for the treatment of skin infections clinically (Bidossi et al. 2019; Bortolin et al. 2017). However, whether CQ has a broad spectrum of anti-inflammatory activity, and its mechanism of action are still unknown. Although it has been documented that CQ can improve psoriasis like dermatitis induced by imiquimod by blocking the activation of NLRP3 inflammasome (Chen et al. 2022a), whether CQ has a targeting inhibitory effect on NLRP3 inflammasome and what are the key groups by which CQ exerts its inhibitory effect, and whether CQ exhibits therapeutic effects in multiple NLRP3-driven diseases have not been elucidated. Therefore, this study focuses on the ability of CQ to specifically inhibit NLRP3 inflammasome activation in human and murine derived macrophages, and evaluates the ameliorative effects of CQ in the model of LPS-induced peritonitis, dextran sodium sulfate (DSS)-induced colitis, and monosodium urate (MSU)-induced gouty arthritis in mice. It would be realistic to expect such a process to deepen the connotation of drug repurposing, expand new indications of CQ in addition to the original indications, and promote the therapeutic use of CQ in multiple NLRP3-driven diseases.

## Materials and methods

### Mice

Six–8-week-old C57BL/6J mice were obtained from Chengdu Yaokang Biotechnology Co., LTD. (Chengdu, China). The mice were specific pathogen-free and maintained on a strict 12-h light/dark conditions with unrestricted access to food and water during the experiments except for specific requirements. All animal experiment protocols were approved by the Animal Care Committee of Chengdu University of Traditional Chinese Medicine (No. 2024024).

### Cell culture

Bone marrow cells were extracted from adult C57BL/6J mice and differentiated into murine bone marrow-derived macrophages (BMDMs) in DMEM medium (Gibco, 11965092) containing 10% fetal bovine serum (FBS, Gibco, 10100), 1% penicillin/streptomycin (Solarbio, P1400). Moreover, murine macrophage colony-stimulating factor (50 ng/mL, MedChemExpress, HY-P7085) was added to the complete growth medium. Human mononuclear leukemia THP-1 cells were grown in RPMI 1640 medium (Gibco, 11875119). To differentiate into macrophages, THP-1 cells were stimulated by phorbol 12-myristate 13-acetate (PMA, 100 nM, MedChemExpress, HY-18739) overnight. All cell lines were cultured under a humidified 5% (v/v) CO_2_ atmosphere at 37 °C.

### Cell viability assays

The cell counting kit-8 (CCK-8) and CellTiter-Glo® luminescent assays were carried out to evaluate the cell viability. Briefly, 1 × 10^5^ cells/well BMDMs were seeded in 96-well growth-medium plate for 24 h. CQ (MedChemExpress, HY-B1360) was diluted with dimethyl sulfoxide (DMSO, MedChemExpress, HY-Y0320), and the cells were treated with CQ for another 24 h. For the CCK-8 assay, the cells were cultured with CCK-8 reagent (MedChemExpress, HY-K0301) for 30 min. The absorbance value at the wavelength of 450 nm were measured. For the CellTiter-Glo® luminescent cell viability assay, the cells were cultured in 96-well white plates and treated with CQ. Then, the CellTiter-Glo reagent (Promega, G7075) was added in each well for 10 min. The luminescence were recorded using Microplate Reader (Tecan, Infinite 200Pro).

### Inflammasome activation

BMDMs at 1 × 10^6^ cells/well or PMA-primed THP-1 macrophages at 8.5 × 10^5^ cells/well were seeded in 12-well plates for 12 h. Next, BMDMs were primed with ultrapure LPS (50 ng/mL, Invivogen, tlrl-3pelps). 4 h later, the complete growth medium in macrophages was changed to serum-free opti-MEM medium (Gibco, 31985070) containing CQ for 1 h. To active canonical NLRP3 inflammasome, nigericin (7.5 μM, Invivogen, tlrl-nig) or ATP (5 mM, Sigma-Aldrich, A2383) was stimulated the cells for 1 h, or transfected with poly(I:C) (2 μg/mL, Invivogen, tlrl-picw), monosodium urate (MSU) crystals (250 μg/mL, Invivogen, tlrl-msu) for 6 h. Poly(dA:dT) (2 μg/mL, Invivogen, tlrl-patn) and ultrapure flagellin (100 ng/mL, Invivogen, tlrl-epstfla) was transfected with Lipo8000™ (Beyotime, C0533) to induce AIM2 and NLRC4 inflammasome activation, respectively. To induce the activation of non-canonical NLRP3 inflammasome, macrophages were stimulated with 1 μg/mL Pam3CSK4 (Invivogen, tlrl-pms), and then 1 μg/mL ultrapure LPS was transfected with Lipo8000™ for 6 h. Cell supernatants and whole cell lysates were collected for the analyses of inflammasome activation.

### Antibodies

Anti-mouse caspase-1 (AG-20B-0042) and Anti-NLRP3 (AG-20B-0014) were from Adipogen. Anti-human cleaved-IL-1β (83186), and Anti-human cleaved-caspase-1 (4199) were bought from Cell Signaling Technology. Mouse IL-1β antibody (AF-401-NA) was from R&D system. Anti-ASC (SC-22514-R) was obtained from Santa Cruz Biotechnology. Anti-GSDMD (ab209845) was from Abcam. GAPDH Monoclonal antibody (60004-1-1 g), Lamin B1 Polyclonal antibody (12987-1-AP), β-actin recombinant antibody (81115-1-RR), Goat Anti-Mouse IgG (SA00001-1), and Goat Anti-Rabbit IgG (SA00001-2) were purchased from Proteintech.

### Caspase-1 activity assay

The Caspase-Glo® 1 Inflammasome assay was obtained from Promega (G9951) to measure caspase-1 activity in supernatants. Briefly, Reconstituting the Caspase-Glo® 1 reagent following the manufacturer’s instructions. Then, the Caspase-Glo® 1 Reagent was added to the cell supernatants to measure luminescence (Tecan, Infinite 200Pro).

### Enzyme-linked immunosorbent assay (ELISA)

Cell supernatants and serum in animal experiments were assayed for mouse IL-1β (Elabscience, E-MSEL-M0003), mouse TNF-α (Elabscience, E-EL-M3063), mouse IL-6 (Dakewe, 1,210,602), and human IL-1β (Dakewe, 1,110,122) in accordance with the manufacturer’s directions.

### Lactate dehydrogenase (LDH) assay

LDH cytotoxicity assay kit (Beyotime, C0016) was used to measure the release of LDH following the manufacturer’s directions. Briefly, LDH cytotoxicity assay kit regent was added to the cell supernatant and incubated for 20 min. The absorbance values were measured at the wavelength of 490 nm in a Microplate Reader (Tecan, Infinite 200Pro).

### ASC oligomerization

The assay for ASC oligomerization has been described previously (Wang et al. 2019b). Brief, cells were lysed by triton buffer for 30 min. 2 mM disuccinimidyl suberate (MedChemExpress, HY-W019543) was added to the resuspended pellets, which were incubated at 30 °C for 30 min. The cross-linked pellets were resuspended in 1 × SDS loading buffer and then boiled for 15 min and analyzed by immunoblot with the anti-ASC antibody.

### Intracellular potassium detection

BMDMs were washed with saline, and then nitric acid was added to the cells. Finally, samples were collected and boiled for 30 min in 100 °C. Inductively coupled plasma mass spectrometry (ICP-MS) was conducted to detect intracellular potassium.

### Reactive oxygen species (ROS) assay

For ROS measurement, BMDMs were loaded with 10 μM DCFH-DA (MedChemExpress, HY-D0940) for 20 min at 37 °C. The cells were resuspended in HBSS after staining and washing. Finally, flow cytometry was conducted to test ROS.

### Immunoprecipitation

BMDMs were stimulated as described above. Then, 200 μL NP-40 lysis buffer (Beyotime, P0013F) containing protease inhibitor was added to lytic the cells for 30 min at 4 °C. The cell lysate was transferred to 1.5 mL EP tubes and centrifuged at 8000 rpm for 10 min at 4 °C. Then, 30 μL cell lysate after centrifugation was added into 30 μL 2 × loading buffer, which was used as whole cell lysate for western blot. Next, protein G beads (MedChemExpress, HY-K0204) are added to the remaining 170 μL cell lysates. Anti-ASC antibody was incubated proportionally and rotated overnight at 4 °C. Subsequently, after cleaning the beads with NP-40 lysis buffer for 5 times, the 1 × loading buffer was added and heated for 15 min at 100 °C for immunoblotting.

### LPS-induced peritonitis mouse model

To evaluate the efficacy of CQ on peritonitis, 6–8-week-old C57BL/6J male mice were injected with CQ (15 and 30 mg/kg, 10 mL/kg) dissolved in saline containing 10% DMSO, 40% PEG 400, and 5% tween-80 for 1 h before injected with LPS [from *Escherichia coli* (O55:B5), Sigma-Aldrich, L2880, 20 mg/kg, 10 mL/kg]. The mice were sacrificed 2 h later. The serum and peritoneal lavage fluids were harvested for the measurement of IL-1β secretion and TNF-α release by ELISA kits. The polymorphonuclear neutrophils (Ly6G^+^CD11b^+^, BioLegend, 127613, 101205) in peritoneal lavage fluids were analyzed by flow cytometry.

### DSS-induced colitis mouse model

DSS is a polyanion derivative, one of the common and effective drugs for inducing ulcerative colitis (Zhang et al. 2022; Chen et al. 2022b). Mouse model of colitis was established with 2.5% (wt/vol) DSS (MP Biomedicals, 9011-18-1) drinking water in 6–8-week-old C57BL/6J male mice for 9 days. Mice were given with CQ (15, 30 mg/kg, 10 mL/kg) by intragastric administration during the model period. Control mice received saline containing 10% DMSO, 40% PEG 400, and 5% tween-80. The weight, consistency of stool and bleeding in stool were recorded every day. The disease activity index (DAI) score, used to access and quantify the severity of intestinal injury during DSS modeling, was counted by the comprehensive results of diarrhea, bloody stool, and the loss of body weight. On the 9th day following induction of colitis, the mice were euthanized, and the serum was collected to detect the content of IL-1β. Colons were removed and washed with PBS, and then the length of colon was measured. Part of colon tissues were put into neutral formalin, and hematoxylin–eosin (HE) staining was conducted to evaluate the pathological injury. The expression of IL-1β in colon tissues was analyzed by immunohistochemical staining. The other colon tissues were collected to detect the inflammasome-related proteins expression.

### MSU-induced gouty arthritis mouse model

To induce gouty arthritis, 6–8-week-old C57BL/6 J male mice were given CQ (15, 30 mg/kg, 10 mL/kg) by intra-articular injection. One hour later, 0.5 mg MSU (Invivogen, tlrl-msu, dissolved in 20 μL PBS) was injected in the ankle joint, and joint size was measured at different time points. After 24 h, the mice were euthanized, and the patella were isolated and cultured in 200 μL opti-MEM medium containing 1% Penicillin–Streptomycin at room temperature for 1 h. The supernatants were collected for ELISA and the joint tissue homogenate were used for western blot analysis.

### Statistical analyses

The experimental data were showed as means ± Standard Error of Mean (SEM). The significant differences were statistically assessed using an unpaired Student’s t-test in two groups or one-way ANOVA followed by Dunnett’s post hoc test in multiple groups. The difference was considered statistically significant when P < 0.05.

## Results

### CQ inhibits caspase-1 activation and IL-1β maturation in mouse BMDMs and human THP-1 macrophages

**Fig. 1:**
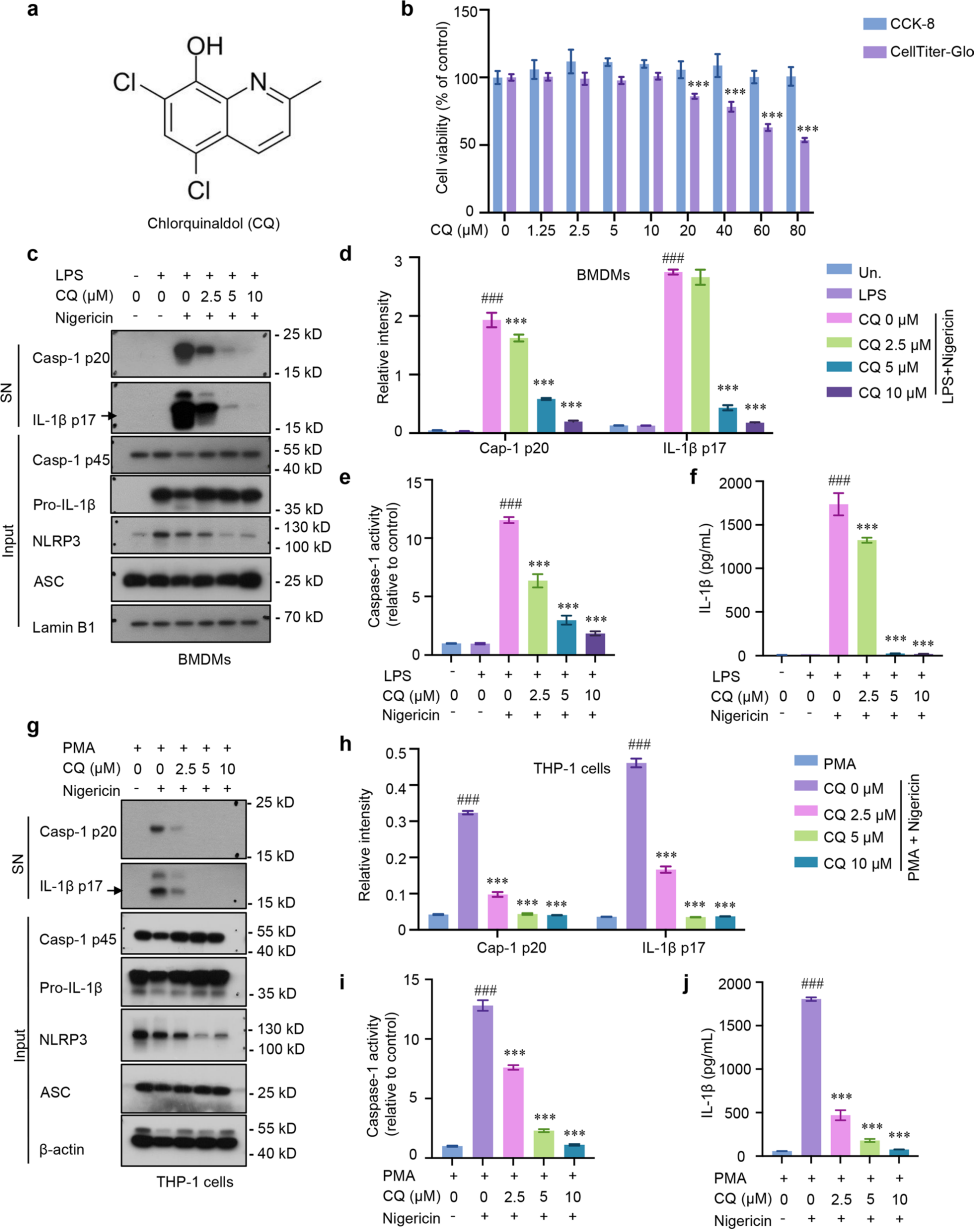
CQ inhibits caspase-1 cleavage and IL-1β secretion in mouse and human macrophages **a** CQ’s structure. **b** Cell viability of CQ in CCK-8 and CellTiter-Glo assays. ^***^P < 0.001 vs. the control group. **c** Immunoblotting analysis of cleaved IL-1β p17, active caspase-1 p20 in supernatants (SN) and pro-IL-1β, caspase-1 p45, NLRP3, ASC in lysates (Input). **d** Relative intensity of caspase-1 p20 and IL-1β p17 described in **c**. **e**, **f** Recombinant luciferase caspase-1 activity (**e**), IL-1β (**f**) production in SN. **g** PMA-primed THP-1 macrophages were stimulated with nigericin with or without CQ. Immunoblotting analysis of cleaved IL-1β p17, active caspase-1 p20 in SN and pro-IL-1β, caspase-1 p45, NLRP3, ASC in input. **h** Relative intensity of caspase-1 p20 and IL-1β p17 described in **g**. **i**, **j** Recombinant luciferase caspase-1 activity (**i**), secretion of IL-1β (**j**) in SN from PMA-primed THP-1 macrophages triggered with nigericin with or without CQ treatment. Statistics differences were analyzed using one-way ANOVA followed by Dunnett’s post hoc test: ^###^P < 0.001 vs. the group of LPS/PMA. ^***^P < 0.001 vs. the group of LPS/PMA + nigericin

To evaluate the regulatory effect of CQ (Fig. 1a) on caspase-1 activation and IL-1β maturation, we first explored the cytotoxicity of CQ in BMDMs. Cell counting kit-8 (CCK-8) and CellTiter-Glo were initially performed to assess CQ on cell viability. As shown in Fig. 1b, pre-treatment with CQ had no obvious cytotoxicity in BMDMs when the concentration was below 10 μM. Next, BMDMs were primed with LPS before treated with CQ, and then provoked with nigericin, an activator of NLRP3 inflammasome. As shown in Fig. 1c, western blotting results exhibited that LPS-primed macrophages expressed high levels of NLRP3 and pro-IL-1β compared with no LPS treatment group. The effect of CQ was confirmed as it dramatically blocked the cleavage of caspase-1 and IL-1β to their mature forms, caspase-1 p20 and IL-1β p17, respectively (Fig. 1c, d). Furthermore, caspase-1 activity in supernatants was strengthened stimulated with nigericin but effectively inhibited by CQ in a concentration-dependent manner (Fig. 1e). Notably, the ELISA results demonstrated that treatment of LPS-primed BMDMs with CQ prior to activation of NLRP3 inflammasome with nigericin led to a dose-dependent reduction in the secretion of IL-1β (Fig. 1f). Consistently, nigericin-induced caspase-1 activity, caspase-1 activation and IL-1β release in PMA-primed THP-1 macrophages could also be obviously impaired by CQ (Fig. 1g–j). Taken together, these results indicate that CQ has the capacity to suppress caspase-1 cleavage and IL-1β maturation in mouse BMDMs and human THP-1 macrophages.

### CQ blunts multiple agonists-induced NLRP3 inflammasome activation and assembly

**Fig. 2:**
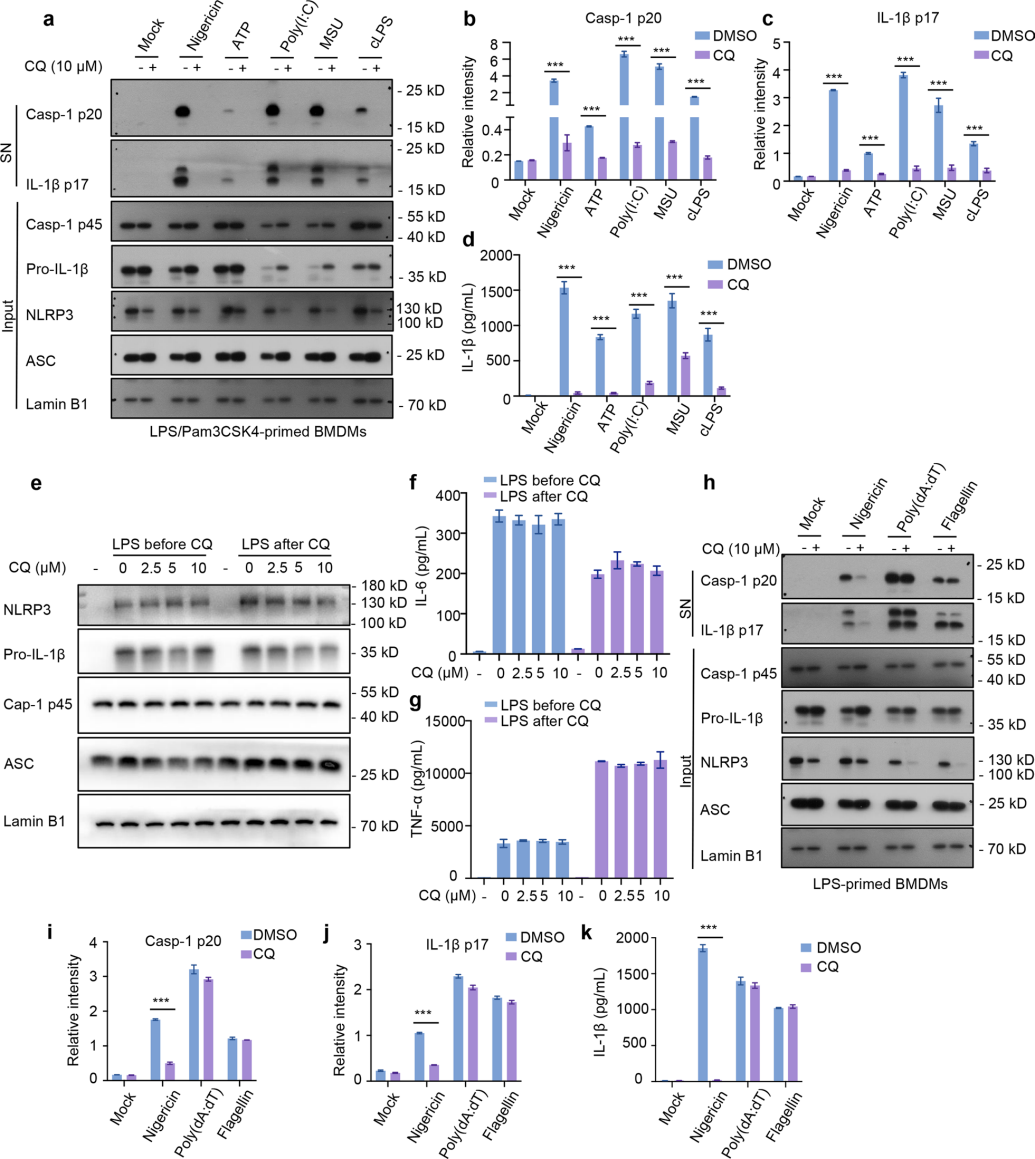
CQ inhibits canonical/noncanonical NLRP3 activation, but does not inhibit NLRC4, AIM2 **a** Immunoblotting analysis of cleaved IL-1β p17, active caspase-1 p20 in SN and pro-IL-1β, caspase-1 p45, NLRP3, ASC in input. **b**, **c** Relative intensity of caspase-1 p20 (**b**) and IL-1β p17 (**c**) described in **a**. **d** IL-1β production in SN from BMDMs primed with LPS or Pam3CSK4, and then stimulated with various stimuli in the presence or absence of CQ. **e** Immunoblotting analysis of pro-IL-1β, caspase-1 p45, NLRP3, ASC in input from BMDMs treated with LPS for 4 h and left stimulated with CQ for 1 h (CQ after LPS) or treated with CQ for 1 h and then stimulated with LPS for 4 h (CQ before LPS). **f**, **g** IL-6 (**f**) and TNF-α (**g**) secretion in SN from BMDMs described in **e**. **h** LPS-primed BMDMs were treated with CQ and then stimulated with nigericin, poly (dA: dT), and flagellin. Immunoblotting analysis of cleaved IL-1β p17, active caspase-1 p20 in SN and pro-IL-1β, caspase-1 p45, NLRP3, ASC in input. **i**, **j** Relative intensity of caspase-1 p20 (**i**) and IL-1β p17 (**j**) described in **h**. **k** IL-1β production in SN from LPS-primed BMDMs stimulated with various activators. Statistics differences were analyzed using an unpaired Student’s t-test in two groups or one-way ANOVA followed by Dunnett’s post hoc test in multiple groups: ^***^P < 0.001 vs. the group of LPS + activators

Activation of NLRP3 inflammasome is strictly regulated to avoid immoderate activation. Aside from nigericin, both PAMPs and DAMPs, including ATP, MSU, poly(I:C), can activates NLRP3 inflammasome. To further investigate the suppression effect of CQ on the activation of NLRP3 inflammasome, the effect of CQ was analyzed in LPS-primed and NLRP3 agonists stimulated BMDMs. As shown in Fig. 2a, caspase-1 p20 and mature IL-1β p17 could be monitored in the supernatants upon ATP, MSU and poly(I:C) stimulation. Similar to nigericin, cleavage of IL-1β and caspase-1 were detected after CQ treatment in BMDMs stimulated with LPS followed by these examined agonists (Fig. 2a–c). Furthermore, we also found that CQ (10 μM) significantly abolished these agonists-induced IL-1β release (Fig. 2d). These results suggest that CQ is a powerful and broad NLRP3 inhibitor.

Intracellular LPS is sensed by a noncanonical NLRP3 inflammasome pathway, which induces caspase-11-dependent IL-1β secretion (Moretti et al. 2022). BMDMs were pretreated with Pam3CSK4 and transfected with ultrapure LPS, and the data exhibited that CQ significantly inhibited caspase-11-dependent caspase-1 activation and IL-1β maturation (Fig. 2a–d), indicating that CQ also blocks cytosolic LPS-induced noncanonical NLRP3 activation. In summary, these data reminder that CQ elicits an inhibitory response to canonical and noncanonical NLRP3 inflammasome activation.

### CQ specifically blocks NLRP3 activation, but not AIM2 or NLRC4

It was vital to confirm that any effect of CQ was specific to NLRP3 activation rather than priming. We then detected whether CQ could affect the priming phase of inflammasome activation by suppressing the expression of NLRP3 or pro-IL-1β protein. The results showed that when BMDMs were treated with CQ before or after LPS priming, CQ had no influence on NLRP3 and pro-IL-1β expression (Fig. 2e), as well as the release of TNF-α, IL-6 in supernatants (Fig. 2f, g). These results elucidate that the blocking action of CQ on NLRP3 inflammasome activation was not regulated the expression of NF-κB-mediated inflammasome precursor proteins.

In order to elucidate whether CQ was specific to the NLRP3 inflammasome or more general, we assessed CQ in AIM2 and NLRC4 inflammasome assays. The results showed that CQ did not blunt caspase-1 cleavage and IL-1β secretion in response to ultrapure flagellin administration (Fig. 2h–k). The effect of CQ on cytosolic double-stranded DNA-induced AIM2 inflammasome was detected by transfecting BMDMs with poly (dA:dT), and the data exhibited that CQ did not block the caspase-1 activation, IL-1β production (Fig. 2h–k). Sum up, these data suggest that CQ can specifically suppress NLRP3 inflammasome activation.

### CQ prevents caspase-1-dependent GSDMD cleavage and pyroptosis in human and mouse macrophages

**Fig. 3:**
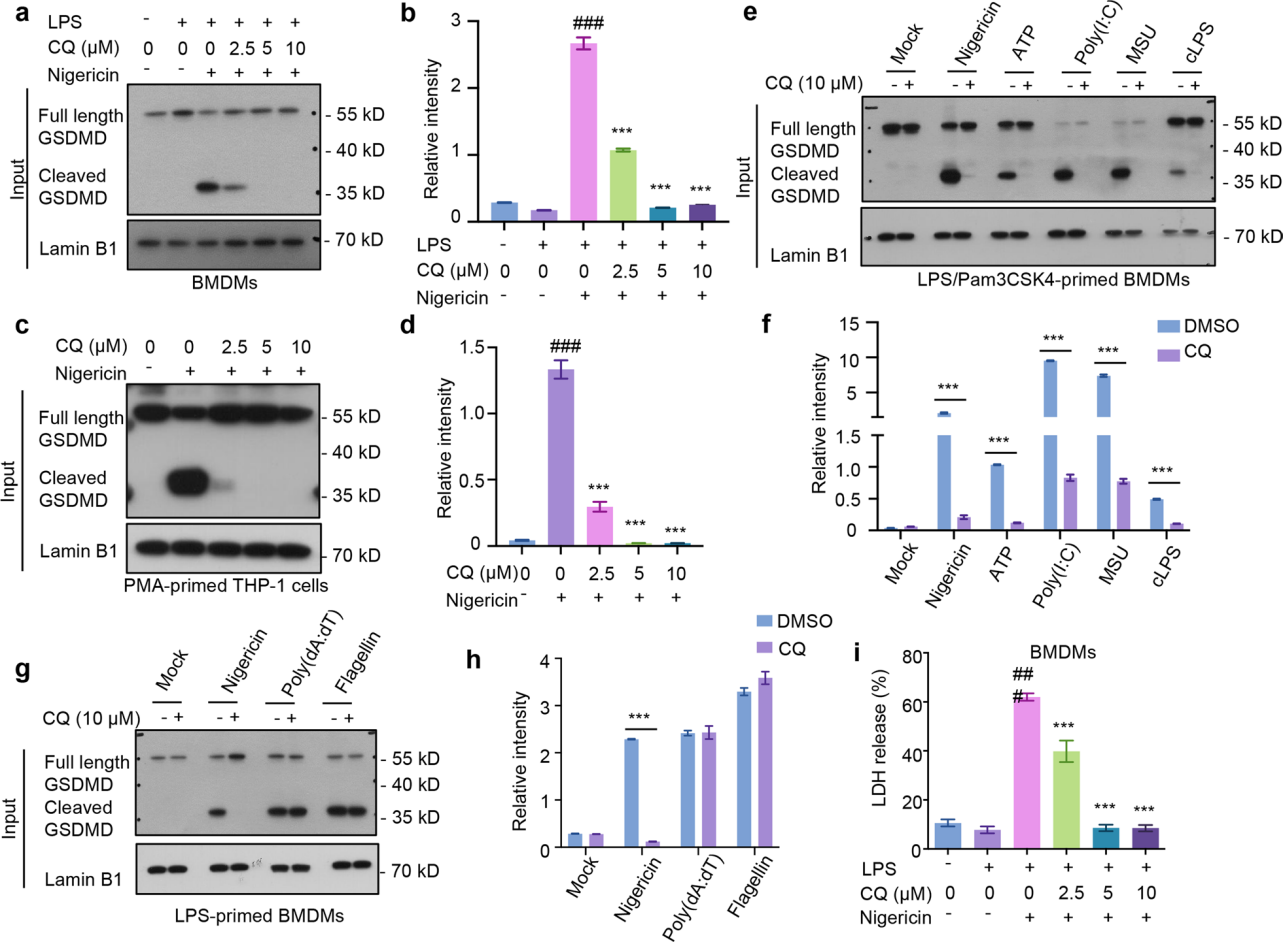
CQ prevents caspase-1-dependent GSDMD cleavage and pyroptosis in human and mouse macrophages **a** LPS-primed BMDMs were stimulated with nigericin after CQ treatment. Western blot analysis of cleaved GSDMD in input. **b** Relative intensity of cleaved GSDMD described in **a**. **c** PMA-primed THP-1 macrophages were stimulated with nigericin after treated with CQ. Immunoblotting analysis of cleaved GSDMD in input. **d** Relative intensity of cleaved GSDMD described in **c**. **e** LPS-primed BMDMs were treated with CQ before stimulated with nigericin, ATP, poly(I:C), MSU, or Pam3CSK4-primed BMDMs were treated with CQ and then stimulated with ultrapure LPS. Immunoblotting analysis of cleaved GSDMD in input. **f** Relative intensity of cleaved GSDMD described in **e**. **g** LPS-primed BMDMs were treated with CQ and then stimulated with nigericin, poly (dA: dT), and flagellin. Western blot analysis of cleaved GSDMD in input. **h** Relative intensity of cleaved GSDMD described in **g**. **i** LDH release in SN from BMDMs. Statistics differences were analyzed using an unpaired Student’s t-test in two groups or one-way ANOVA followed by Dunnett’s post hoc test in multiple groups: ^###^p < 0.001 vs. the group of LPS, ^***^P < 0.001 vs. the group of LPS + activators

Pyroptosis is a proinflammatory cell death that is correlative with pathogenesis of various chronic inflammatory diseases. GSDMD was identified as a cleavage target of caspase-1 required for pyroptosis and IL-1 secretion (Shi et al. 2015). We then detected whether CQ could reduce the cleaved GSDMD. The results demonstrated that caspase-1 was cleaved and activated after stimulated by nigericin in LPS-primed BMDMs and PMA-primed THP-1 macrophages, leading to the downstream cleavage of GSDMD and pyroptosis (Fig. 3a–d). Notably, cleaved GSDMD in the CQ-treated group was significantly blocked compared to that in the nigericin-treated group in a concentration-dependent manner (Fig. 3a–d). Similarly, a sustained decrease in cleaved GSDMD levels stimulated by various NLRP3 activators (nigericin, ATP, poly(I:C), MSU, and cLPS) was observed after CQ treatment (Fig. 3e, f). Then, we investigated whether NLRC4 or AIM2 is involved in macrophage pyroptosis induced by CQ and found that there was no change in cleaved GSDMD after CQ treatment (Fig. 3g, h), supporting that it is the canonical and non-canonical NLRP3 that mediates caspase-1-dependent GSDMD cleavage and pyroptosis. LDH release in supernatants was examined as a readout for pyroptosis. A test of the supernatant LDH showed that nigericin induced LDH release in LPS-primed BMDMs, while CQ markedly blocked nigericin-induced LDH release (Fig. 3i), suggesting that the negative role of CQ on cell death in BMDMs. Altogether, these results suggest that CQ is a superior caspase-1 inhibitor that inhibits GSDMD-dependent pyroptosis by preventing NLRP3 inflammasome-mediated caspase-1 activation.

### CQ inhibits NLRP3-mediated ASC oligomerization, but does not suppress potassium efflux or the production of ROS

**Fig. 4:**
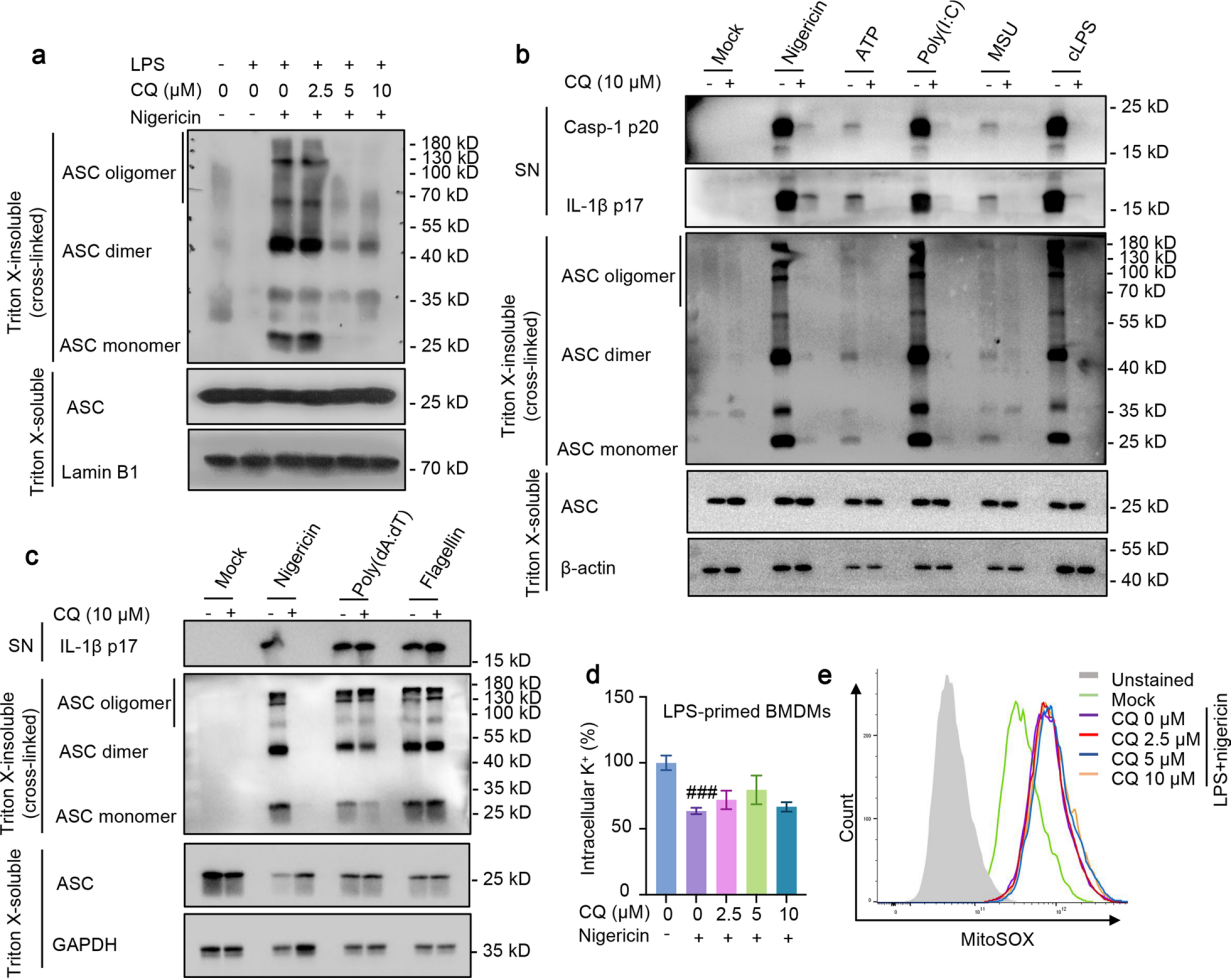
CQ inhibits ASC oligomerization, but does not suppress potassium efflux or the production of ROS. **a** LPS-primed BMDMs were treated with CQ and then stimulated with nigericin. Immunoblotting analysis of cleaved IL-1β p17, active caspase-1 p20 in SN, and ASC in Input and cross-linked cytosolic pellets. **b** Immunoblotting analysis of cleaved IL-1β p17, active caspase-1 p20 in SN, and ASC in input and cross-linked cytosolic pellets of LPS/Pam3CSK4-primed BMDMs treated with CQ and then stimulated with various stimuli. **c** Immunoblotting analysis of cleaved IL-1β p17, active caspase-1 p20 in SN, and ASC in Input and cross-linked cytosolic pellets of LPS-primed BMDMs treated with CQ and then stimulated with nigericin, poly (dA:dT), flagellin. **d** LPS-primed BMDMs were treated with CQ and then stimulated with nigericin, and then HNO_3_ was added to lyse BMDMs. Intracellular potassium detection was performed by ICP-MS. **e** LPS-primed BMDMs were stimulated with nigericin after CQ treatment. Then, BMDMs were loaded with 10 μM DCFH-DA. Flow cytometry were conducted to test ROS. Statistics differences were analyzed using one-way ANOVA followed by Dunnett’s post hoc test in multiple groups: ^###^p < 0.001 vs. the group of LPS

Since ASC oligomerization plays a critical role in the recruitment and subsequent activation of caspase-1, we further investigated whether CQ mediates NLRP3 inflammasome activation by inhibiting ASC oligomerization. Cytosolic fractions from cell lysates were cross-linked, and we observed higher levels of ASC oligomerization after LPS and nigericin co-treatment in BMDMs, while CQ perturbed ASC-complex formation in a concentration-dependent manner (Fig. 4a). Further findings revealed that CQ blocked ASC oligomerization, caspase-1 cleavage and IL-1β production after treatment with NLRP3 inflammasome stimulation, including ATP, nigericin, poly(I:C), MSU and intracellular LPS (Fig. 4b), confirming that CQ blunts NLRP3 activation by impairing ASC oligomerization. These data suggest that CQ may act directly on ASC oligomerization or involve in its upstream events to exert effects during assembly.

ASC oligomerization is essential for NLRP3 and AIM2 activation but may not be required for the activation of NLRC4 inflammasome. Additionally, we quested whether the inhibition effect of NLRP3 inflammasome activation by CQ was due to direct target suppression of ASC oligomerization. In line with the effect of CQ on the flagellin and poly(dA:dT)-induced IL-1β processing, ASC oligomerization was not affected by CQ under the stimulation of NLRC4 and AIM2 inflammasome agonists, flagellin and poly (dA:dT), respectively (Fig. 4c). These data further prove that CQ exerts its inhibitory effect by acting upstream of ASC oligomerization.

To make further efforts clarify the biological mechanism by which CQ affects NLRP3 inflammasome activation, we measured the effect of CQ on K^+^ efflux, a common upstream trigger of NLRP3 inflammasome activation (He et al. 2016). Notably, when LPS initiated BMDMs were stimulated with nigericin, the intracellular potassium dramatically decreased, but the change was not reversed by CQ treatment (Fig. 4d). In addition, mitochondrial ROS production is known to trigger NLRP3 inflammasome assembly (Zhou et al. 2011). We examined and found that CQ did not affect nigericin-induced ROS (Fig. 4e). Together, these results demonstrate that CQ’s repression effect on NLRP3 inflammasome activation is independent of both K^+^ efflux and ROS.

### CQ inhibits inflammasome assembly by blocking NLRP3-ASC interaction, and hydroxyl on the benzene ring contributes to the inhibiting action of CQ

**Fig. 5:**
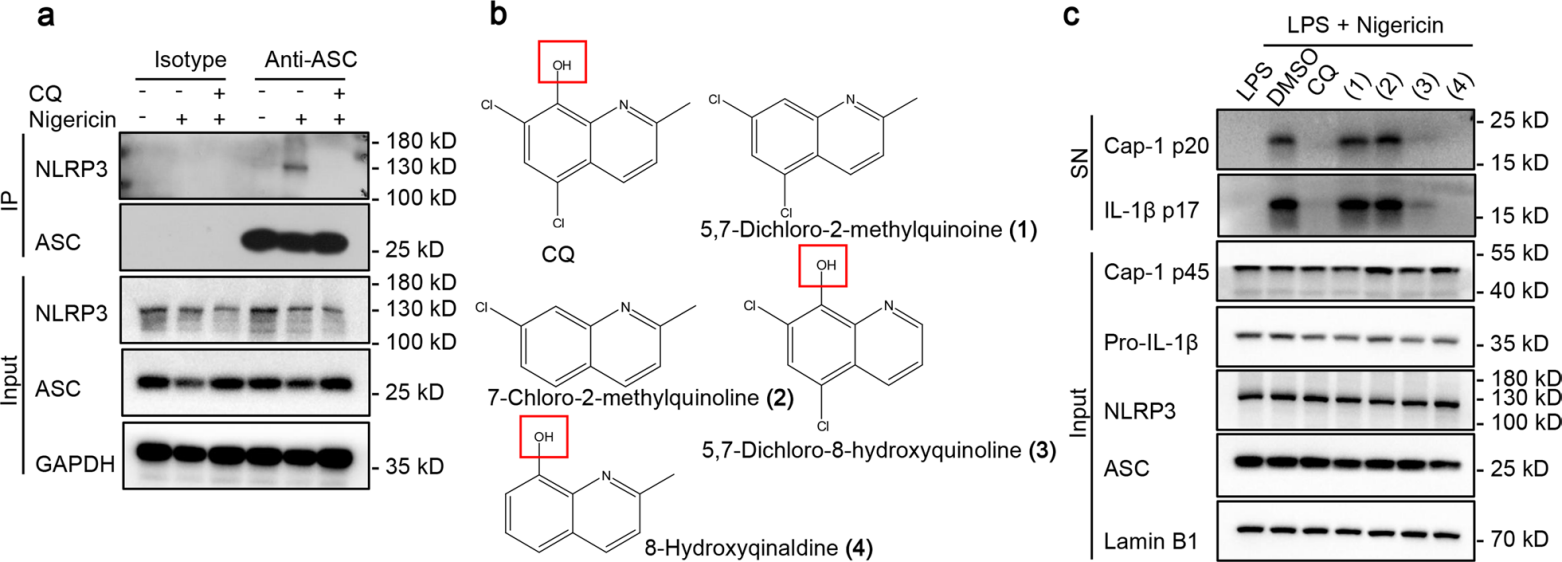
CQ blocks NLRP3-ASC interaction, and hydroxyl on the benzene ring contributes to the inhibiting action of CQ **a** LPS-primed BMDMs were treated with CQ and then stimulated with nigericin. Protein G beads are added to the cell lysates. Anti-ASC antibody was incubated proportionally and rotated overnight. Immunoblotting analysis of NLRP3 and ASC in input. **b** the structure of CQ derivative. **c** LPS-primed BMDMs were treated with CQ or CQ derivative, and then stimulated with nigericin. Immunoblotting analysis of cleaved IL-1β p17, active caspase-1 p20 in SN and pro-IL-1β, caspase-1 p45, NLRP3, ASC in input

We next assessed the effects of CQ on the formation NLRP3 inflammasome. An important step in NLRP3 inflammasome assembly is the interplay between NLRP3 and ASC. Therefore, we investigated whether CQ could block the formation of the endogenous NLRP3 inflammasome complex induced by nigericin, and the results of immunoprecipitation assay showed that CQ inhibited endogenous NLRP3-ASC interaction (Fig. 5a), which indicated that CQ plays a key role by suppressing NLRP3-ASC interaction. We then sought to determine the key functional group in CQ responsible for the inhibitory effect on NLRP3 inflammasome. Different CQ derivatives were selected to evaluate their inhibitory effect on caspase-1 cleavage and IL-1β secretion (Fig. 5b). Interestingly, nigericin-induced caspase-1 activation and IL-1β release could be obviously blocked by 5,7-dichloro-8-hydroxyquinoline (3) and 8-hydroxyqinaldine (4) (Fig. 5c). Surprisingly, treatment of LPS-primed BMDMs with 5,7-dichloro-2-methylquinoine (1) and 7-chloro-2-methylquinoline (2) prior to activation of NLRP3 inflammasome with nigericin did not result in a reduction in caspase-1 cleavage and IL-1β secretion (Fig. 5c). It can be seen that the hydroxyl on the benzene ring is essential for CQ-mediated inhibition of NLRP3 inflammasome.

### CQ treatment ameliorates LPS-induced peritonitis in vivo

**Fig. 6:**
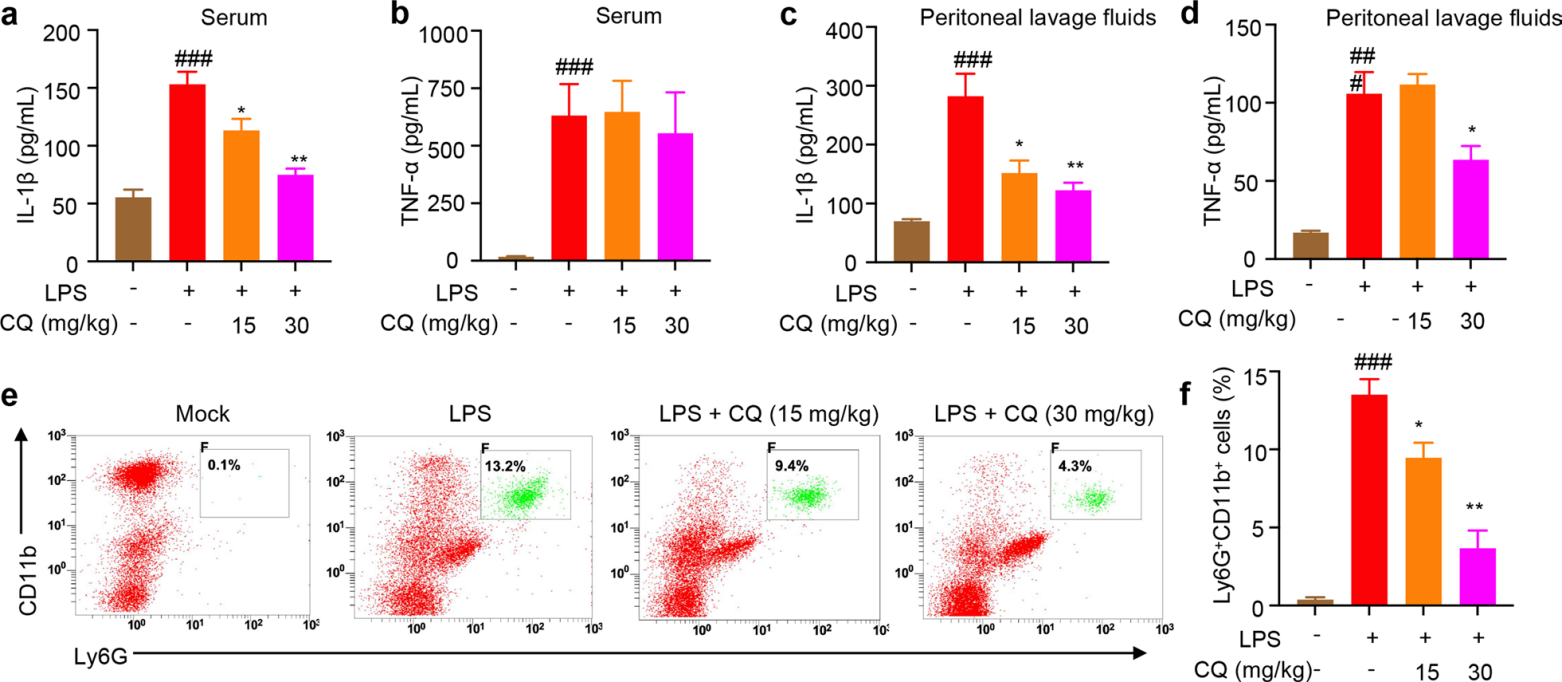
CQ ameliorates LPS-induced peritonitis **a**–**d** Serum levels of IL-1β (**a**), TNF-α (**b**) and peritoneal lavage fluids IL-1β (**c**), TNF-α (**d**) as measured by ELISA. **e**, **f** The proportion of neutrophils (Ly6G^+^CD11b^+^) in peritoneal lavage fluids were analyzed by flow cytometry. Statistics differences were analyzed using one-way ANOVA followed by Dunnett’s post hoc test in multiple groups: ^###^P < 0.001 vs. the control, ^*^P < 0.05, ^**^P < 0.01 vs. the group of LPS

We then verified the role of CQ on NLRP3 inflammasome activation in vivo. The induction of IL-1β by intraperitoneal injection of LPS is shown to be NLRP3 dependent (Li et al. 2022; Rathinam et al. 2019). We further evaluated the efficacy of CQ in a mouse model of LPS-induced peritonitis. In our experiments, after 2 h injection of LPS, IL-1β secretion in the mice serum and peritoneal lavage fluid was significantly increased compared to the control group (Fig. 6a, c). In vivo production of IL-1β were completely eliminated in CQ treatment mice after LPS injection (Fig. 6a, c). The data show that pretreatment with CQ did not considerably attenuate LPS-induced serum TNF-α production (Fig. 6b), indicating that CQ is active in vivo. This ameliorated septic response in CQ/LPS-injected mice was further emphasized by the significant reduction of serum and peritoneal lavage fluid TNF-α levels (Fig. 6d), which is a key marker of sepsis. In addition, consistent with the inhibitory effects of CQ on proinflammatory cytokines, the proportion and the number of neutrophils in peritoneal lavage cells were also reduced in CQ treatment mice (Fig. 6e, f). This results clearly indicated that CQ ameliorated LPS-mediated peritonitis and cytokines release in vivo.

### CQ exhibits a therapeutic effect in DSS-induced colitis model

**Fig. 7:**
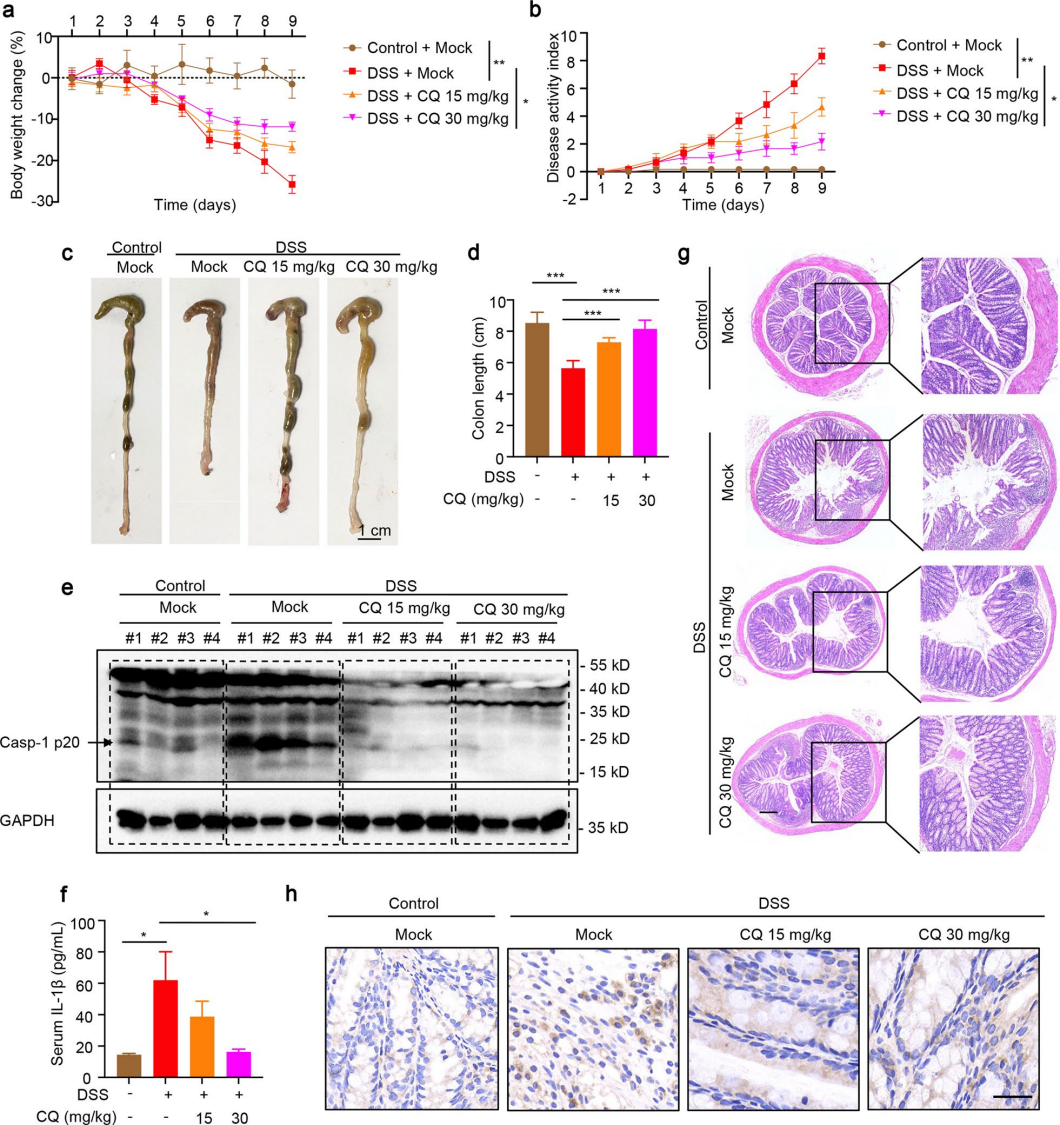
CQ exhibits a therapeutic effect in DSS-induced colitis model. **a**, **b** Body weight change (**a**) and DAI score (**b**) were evaluated (n = 6 mice for each group). **c**, **d** Representative colon image (**c**) and the colon lengths (**d**) were measured after DSS or DSS plus CQ treatment. Scale bar: 1 cm. **e** Western blot analysis of active caspase-1 p20 in colon tissues after homogenization of protein content. **f** ELISA analysis of serum IL-1β production of DSS-fed mice with vehicle or CQ treatment for 9 days. **g**, **h** HE staining (Scale bar: 200 μm) (**g**) and immunohistochemical staining (Scale bar: 20 μm) of IL-1β (**h**) in colon sections were measured 9 days after treatment with DSS plus vehicle or CQ. Statistics differences were analyzed using one-way ANOVA followed by Dunnett’s post hoc test in multiple groups: ^*^P < 0.05, ^**^P < 0.01, ^***^P < 0.001 vs. the group of DSS

DSS-induced colitis in mice is an experimental NLRP3 inflammasome-related inflammation model. Targeting inhibition of NLRP3 inflammasome has been shown to have clear therapeutic effects in the aforementioned mouse model (Bauer et al. 2010; Xu et al. 2021). In the current experiment, this colitis model was used to verify the inhibitory effect of CQ on NLRP3 inflammasome activation in vivo. The results exhibited that mice developed severe colitis characterized by diarrhea, intestinal bleeding, body weight loss and shortened colon length after receiving 2.5% DSS drinking water. Compared with the DSS model group, the weight loss of mice in the CQ group was significantly attenuated, and the colon length became longer (Fig. 7a, c, d). The DAI was maximized toward the end of 9 days of 2.5% DSS administration, which reflected the severity of the colitis, however, CQ prevented this increase in DAI (Fig. 7b). To validate whether CQ affects NLRP3 inflammasome activation in the experimental colitis model, cytokines secretion and proteins expression were analyzed in this study. As expected, CQ intervention significantly attenuated caspase-1 activation in the colon tissue of DSS-treated mice (Fig. 7e). Moreover, ELISA results revealed that serum IL-1β production was dramatically blocked by administering CQ to mice with DSS-induced colitis (Fig. 7f). Histological results showed that after DSS modeling, the colon of mice developed severe pathological damages, including goblet cells loss, crypts distortion, and neutrophils and monocytes filtration. Astonishingly, the oral administration of CQ was associated with a significant reduction in the severity of colitis (Fig. 7g). Immunohistochemical staining also showed that CQ could significantly inhibit the expression of IL-1β in colon tissues (Fig. 7h). Summary, CQ exerts an excellent ameliorating effect on the DSS-induced NLRP3 inflammasome-related colitis.

### CQ relieves the symptoms of MSU-induced gouty arthritis

**Fig. 8:**
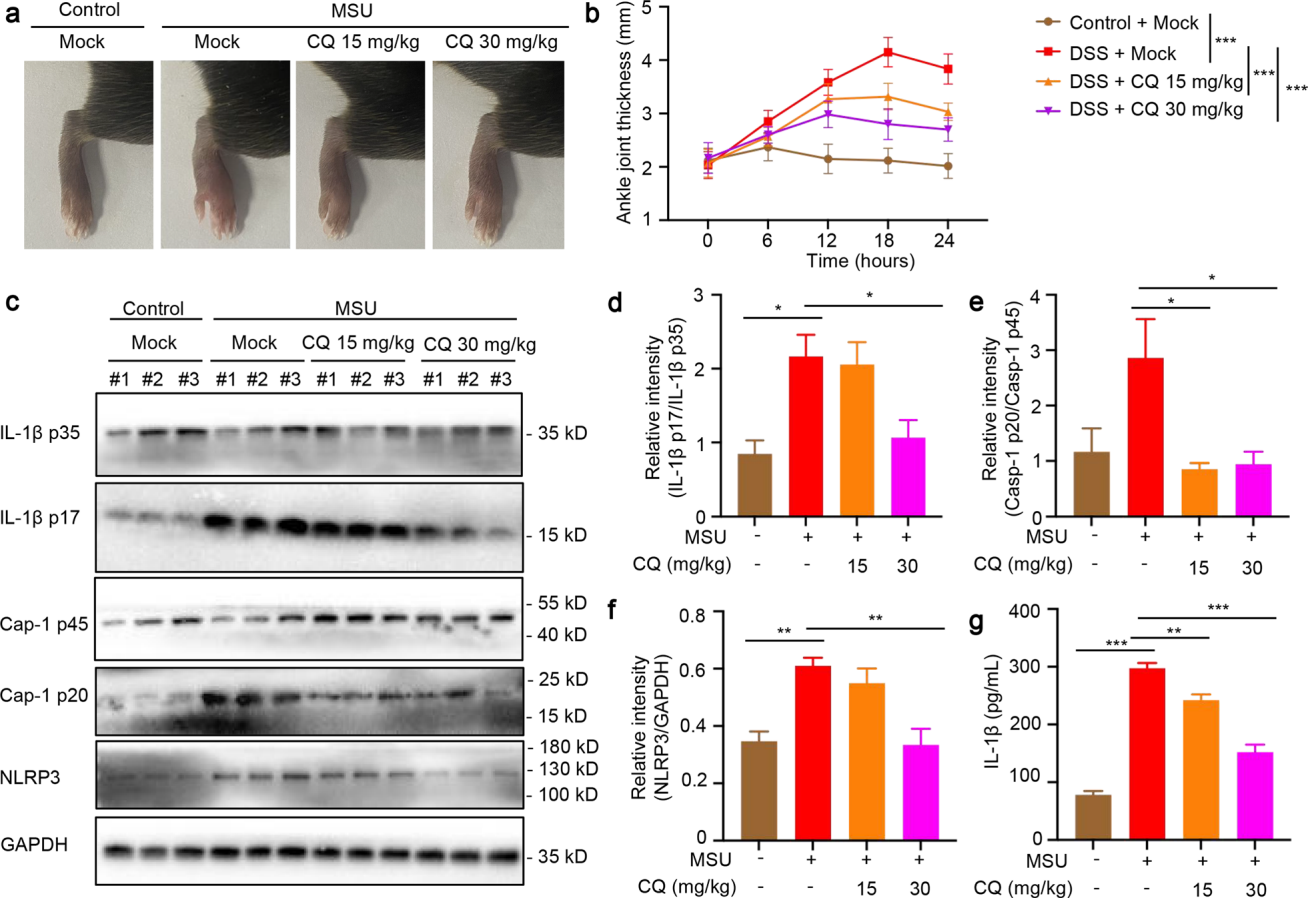
CQ relieves the symptoms of MSU-induced gouty arthritis. **a**, **b** Representative images of ankle joint thickness taken 24 h after MSU injection (**a**). Measurement of the ankle joint thickness of each mouse at different time points after MSU injection (**b**). **c** Western blot analysis of IL-1β p35, IL-1β p17, caspase-1 p45, caspase-1 p20, and NLRP3 in the joint homogenate. **d**–**f** Relative intensity of IL-1β p17 (**d**), caspase-1 p20 (**e**), and NLRP3 (**f**) described in **c**. **g** ELISA analysis of serum IL-1β production in the washing fluid of ankle joints. Statistics differences were analyzed using one-way ANOVA followed by Dunnett’s post hoc test in multiple groups: ^*^P < 0.05, ^**^P < 0.01, ^***^P < 0.001 vs. the group of MSU

Joint inflammation induced by MSU crystals is dependent on NLRP3 inflammasome. IL-1β is the major effector cytokine produced in gout. To investigate CQ's role in inflammatory joint disease, we used the well-established MSU-induced gouty arthritis mouse model. The results showed that when MSU crystals were injected into the ankle joints of mice, a significant increase in ankle swelling was observed. Interestingly, oral CQ dose-dependently reduced ankle swelling (Fig. 8a, b). Western blot analysis indicated that MSU crystals could induce the expression of IL-1β p17 and NLRP3, and CQ treatment significantly attenuated the expression of IL-1β p17, caspase-1 p20 and NLRP3 in the joint homogenate of MSU-treated mice (Fig. 8c–f). In addition, the significantly higher level of IL-1β in the washing fluid of ankle joints after injection of MSU crystals was abolished by administration of CQ (Fig. 8g). Taken together, these results suggest that CQ inhibits NLRP3 inflammasome activation and IL-1β secretion in vivo in the context of MSU-induced gouty inflammation.

## Discussion

At present, breakthroughs have been made in the drug repurposing. For example, the biguanide oral hypoglycemic drug metformin, which is used to treat type 2 diabetes, is widely used in clinical practice as a first-line drug. Its application has expanded to multiple indications, such as metastatic breast cancer, fragile X syndrome, and aging (Wang et al. 2019c; Gantois et al. 2019; Kulkarni et al. 2020; Pernicova and Korbonits 2014). As a highly toxic mineral traditional Chinese medicine, arsenic has a significant effect on hemorrhoids, scrofula, carbuncle, acne, tinea, malaria. Now arsenic (arsenic trioxide) has become a good medicine for the treatment of acute promyelocytic leukemia (APL) (Kutny et al. 2022; Sanz et al. 2019). Moreover, its treatment spectrum is increasingly wide, and it has obvious effects on hepatocellular carcinoma, metastatic melanoma, malignant lymphoma and various solid tumors (Chen et al. 2023; Fang and Zhang 2020; Sönksen et al. 2022; Zhang et al. 2021). The development of completely new drugs is often more step-by-step, long cycle time and high cost, while the safety of existing drugs is generally good, and the research data and clinical data are abundant. The drug repurposing as an effective strategy in drug development can shorten the research cycle, reduce costs, reduce risks, and improve outputs (Parvathaneni et al. 2019). In our experiment, the antimicrobial agent CQ showed a potent inhibitory effect on NLRP3 inflammasome in human and mouse macrophages. Importantly, CQ has no effect on the activation of AIM2 or NLRC4 inflammasome, indicating the specifically inhibitory effect of CQ. More importantly, administration of CQ has remarkable therapeutic effects on LPS-induced peritonitis, DSS-induced colitis, and MSU-induced gouty inflammation in mice, which provides new opportunities for the development of new indications for CQ.

In an exaggerated immune response, neutrophils, monocytes, macrophages and other inflammatory cells trigger a cascade reaction of multiple cytokines through a specific positive feedback regulatory mechanism, and the uncontrolled rise of cytokines in tissues and organs. Overactivated cytokines mediate the occurrence of cytokine storms by modulating the immune response. Cytokine storms play a key role in driving the transformation of many diseases into severe ones. The SARS coronavirus, avian influenza virus, and SARS-CoV-2 can cause cytokine storms, which in turn can lead to severe acute lung injury (ALI), acute respiratory distress syndrome (ARDS), septic shock, and even death in critically ill patients due to multiple organ failure (Hu et al. 2021; Mangalmurti and Hunter 2020; Ye et al. 2020). IL-1β, as an "early response cytokine", can promote the activation and release of pro-inflammatory cytokines and chemokines, and is one of the key pro-inflammatory factors in the formation of cytokine storms. Therefore, regulating the release of IL-1β can be an important strategy and method to control cytokine storms. The NLRP3 inflammasome is a key molecular mechanism that regulates the maturation and secretion of IL-1β. Therefore, targeted inhibition of NLRP3 inflammasome can be an important strategy and method to prevent cytokine storms caused by abnormal activation of NLRP3 inflammasome. The NLRP3 inflammasome plays an important role in innate immune-mediated inflammation, and its aberrant activation is closely associated with several human diseases, such as gout, atherosclerosis, as well as inflammatory bowel diseases.

Current studies have identified a variety of NLRP3 inhibitors, but whether these agents can ultimately be used to treat NLRP3 inflammasome related diseases awaits further clinical observation. Therefore, we still need to find closer to clinical application, more active and safer NLRP3 inhibitors to provide an optimal strategy for the treatment of NLRP3 inflammasome related diseases. In the current experiment, we found that the antibacterial agent CQ is a specific NLRP3 inflammasome inhibitor. On the one hand, CQ acts upstream of ASC oligomerization and inhibits NLRP3 inflammasome activation by blocking the interaction of NLRP3 and ASC. On the other hand, the hydroxyl group on the phenyl ring of CQ plays an important role on the inhibitory effect. In addition, we verified that CQ exhibits obvious protective effects against NLRP3-driven diseases in the model of LPS-induced peritonitis, DSS-induced colitis, and MSU-induced gouty inflammation, and provides new ideas as well as theoretical and practical basis for the prevention and treatment of NLRP3-driven diseases.

## Conclusions

In summary, in this study, we found that CQ is a highly potent and specific inhibitor of NLRP3 inflammasome and has significant therapeutic effects on NLRP3 inflammasome-related inflammatory diseases. It is expected that this process can deepen the connotation of drug repurposing and develop new applications of CQ on the basis of the original indications.

## Data Availability

The datasets used and/or analysed during the current study are available from the corresponding author on reasonable request.
